# Comparative and Combinatorial Effects of Resveratrol and Sacubitril/Valsartan alongside Valsartan on Cardiac Remodeling and Dysfunction in MI-Induced Rats

**DOI:** 10.3390/molecules26165006

**Published:** 2021-08-18

**Authors:** Pema Raj, Karen Sayfee, Mihir Parikh, Liping Yu, Jeffrey Wigle, Thomas Netticadan, Shelley Zieroth

**Affiliations:** 1 Department of Physiology and Pathophysiology, University of Manitoba, Winnipeg, MB R3E 0J9, Canada; praj@sbrc.ca (P.R.); parikhmihirp@outlook.com (M.P.); 2 Canadian Centre for Agri-Food Research in Health and Medicine, Winnipeg, MB R2H 2A6, Canada; karensayfee@gmail.com (K.S.); lyu@sbrc.ca (L.Y.); 3 Agriculture and Agri-Food Canada, Winnipeg, MB R3T 2M9, Canada; 4 Department of Biochemistry and Medical Genetics, University of Manitoba, Winnipeg, MB R3E 0J9, Canada; jwigle@sbrc.ca; 5 Institute of Cardiovascular Sciences, St. Boniface Hospital Albrechtsen Research Centre, Winnipeg, MB R2H 2A6, Canada; 6 Section of Cardiology, Department of Medicine, University of Manitoba, Winnipeg, MB R2H 2A6, Canada

**Keywords:** resveratrol, sacubitril/valsartan, heart failure, myocardial infarction

## Abstract

The development and progression of heart failure (HF) due to myocardial infarction (MI) is a major concern even with current optimal therapy. Resveratrol is a plant polyphenol with cardioprotective properties. Sacubitril/valsartan is known to be beneficial in chronic HF patients. In this study, we investigated the comparative and combinatorial benefits of resveratrol with sacubitril/valsartan alongside an active comparator valsartan in MI-induced male Sprague Dawley rats. MI-induced and sham-operated animals received vehicle, resveratrol, sacubitril/valsartan, valsartan alone or sacubitril/valsartan + resveratrol for 8 weeks. Echocardiography was performed at the endpoint to assess cardiac structure and function. Cardiac oxidative stress, inflammation, fibrosis, brain natriuretic peptide (BNP), creatinine and neutrophil gelatinase associated lipocalin were measured. Treatment with resveratrol, sacubitril/valsartan, valsartan and sacubitril/valsartan + resveratrol significantly prevented left ventricular (LV) dilatation and improved LV ejection fraction in MI-induced rats. All treatments also significantly reduced myocardial tissue oxidative stress, inflammation and fibrosis, as well as BNP. Treatment with the combination of sacubitril/valsartan and resveratrol did not show additive effects. In conclusion, resveratrol, sacubitril/valsartan, and valsartan significantly prevented cardiac remodeling and dysfunction in MI-induced rats. The reduction in cardiac remodeling and dysfunction in MI-induced rats was mediated by a reduction in cardiac oxidative stress, inflammation and fibrosis.

## 1. Introduction

The renin-angiotensin-aldosterone system (RAAS) blockade is unequivocally established as the first and foremost clinically beneficial strategy in post-myocardial infarction (MI) and heart failure (HF) therapies [1]. The natriuretic peptide (NPs) system also plays an important beneficial counter-regulatory role in limiting the detrimental effects of RAAS [2,3]. The maintenance of circulating levels of NPs has been recognized as an effective strategy in HF patients. However, the use of a human recombinant-NP such as nesiritide to achieve this goal has met with limited success in HF patients [4,5]. Thus augmenting the levels of circulating NPs by blocking their degradation via pharmacological agents has become an area of keen interest in the drug therapy for HF [6,7].

Neprilysin is a metalloprotease which degrades NPs, such as atrial natriuretic peptide, C-type natriuretic peptide, brain natriuretic peptide (BNP) and substance P [5]. The manipulation of the NP system to increase the levels of NPs via a neprilysin inhibitor has been proposed as a new therapy. The blockade of neprilysin alone by an inhibitor without RAAS blockade is not an effective treatment strategy as it increases vasoconstriction [5]. The suppression of RAAS while enhancing the NP system via the simultaneous inhibition of both angiotensin converting enzyme (ACE) and neprilysin has been proven less successful and has the risk of angioedema [4,8]. A first-in-class combination drug called sacubitril/valsartan that together inhibits neprilysin and blocks the angiotensin receptor has been discovered as an alternative. The prospective comparison of angiotensin receptor and neprilysin inhibitor with ACE inhibitor to determine impact on global mortality and morbidity in HF (PARADIGM-HF trial), (NCT01035255), demonstrated that treatment with sacubitril/valsartan was superior to the ACE inhibitor, enalapril, in reducing mortality and morbidity in patients with HF with reduced ejection fraction [9]. The findings of the PARADIGM-HF trial support the further pursuit of combination treatment strategies with new potential molecules that can act through different therapeutic targets.

Resveratrol is an extensively studied polyphenol with significant cardioprotective effects via its pleotropic action [10,11]. Our previous study showed that low dose resveratrol was as effective as an ACE inhibitor in preventing cardiac remodeling and dysfunction in MI-induced young male rats [12]. A previous clinical trial also showed that resveratrol improved endothelial function and diastolic function in post-MI patients [13]. It is also important to understand the preclinical efficacy of resveratrol alongside newer HF drugs as it will provide valuable information. Resveratrol along with sacubitril/valsartan may therefore be a promising approach to tackle MI-induced HF development. In this study, we hypothesized that stand-alone and combination treatment with resveratrol and sacubitril/valsartan will be cardioprotective in the setting of MI. Thus, we investigated the efficacy of stand-alone and combinatorial treatment with resveratrol and sacubitril/valsartan in MI-induced male rats.

## 2. Results

### 2.1. General Characteristics

**Figure 1:**
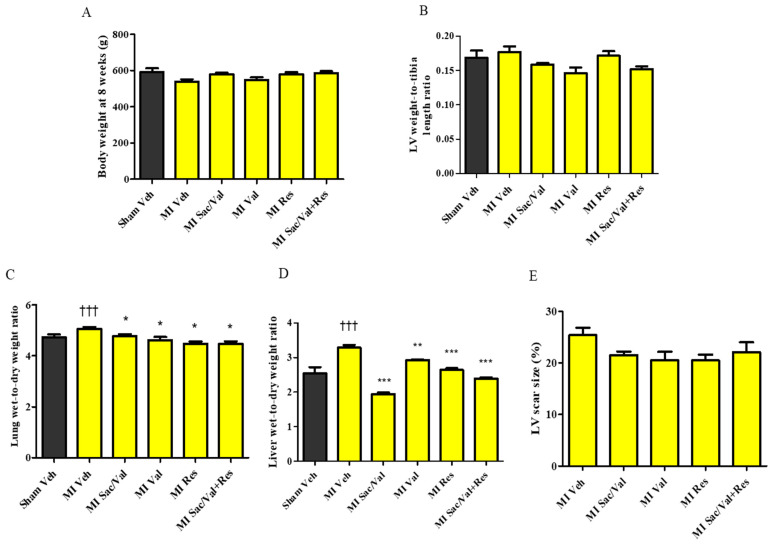
(**A**) Body weight of sham and MI rats at week 8; (**B**) LV weight-to-tibia length ratio of sham and MI rat; (**C**) Lung wet-to-dry weight ratio of sham and MI rats. (**D**) Liver wet-to-dry weight ratio of sham and MI rats. (**E**) LV scar size (%) in MI rats. All values are expressed as mean ± SEM, *n* = 9–11. ^†††^ *p* <0.001 vs. Sham Veh; * *p* < 0.05, ** *p* < 0.01, *** *p* < 0.001 vs. MI Veh. All values are expressed as mean ± SEM. Veh—vehicle, Sac/Val—sacubitril/valsartan, Res—resveratrol, Val—valsartan.

The body weight was comparable between the sham-operated rats and MI-induced rats in all groups at 8 weeks after the surgery (Figure 1A). LV-to-tibia length ratio was also comparable between sham-operated and MI-induced rats in all groups (Figure 1B). In sham-operated and MI-induced rats, pleural and abdominal cavities were devoid of effusions or ascites at 8 weeks. There was a significant increase in lung wet-to-dry weight ratio in vehicle-treated MI-induced rats compared to vehicle-treated sham-operated rats (Figure 1C. 5.07 ± 0.07 vs. 4.73 ± 0.12, *p* < 0.05). Sacubitril/valsartan, valsartan, resveratrol and sacubitril/valsartan + resveratrol significantly lowered lung wet-to-dry weight ratio in MI-induced rats compared to vehicle-treated MI-induced rats (Figure 1C. 5.07 ± 0.07 vs. 4.77 ± 0.82, 4.62 ± 0.12, *p* < 0.05, and 4.47 ± 0.09, *p* < 0.01, and 4.48 ± 0.10, *p* < 0.001).

MI-induced rats treated with vehicle had significantly increased liver wet-to-dry weight ratio compared to sham-operated rats at 8 weeks (Figure 1D. 3.29 ± 0.07 vs. 2.54 ± 0.18, *p* < 0.001). MI-induced rats administered with sacubitril/valsartan, valsartan, resveratrol and sacubitril/valsartan + resveratrol had a significantly lower liver wet-to-dry weight ratio (Figure 1D. 3.29 ± 0.07 vs. 1.95 ± 0.04, *p* < 0.001, 2.93 ± 0.15, *p* < 0.01, 2.65 ± 0.05, *p* < 0.001, and 2.40 ± 0.03, *p* < 0.001) compared to MI-induced rats treated with vehicle. 

All MI-induced rats included in the study had well-defined scarred LV tissue at the anterior region due to the large anterior-infarct that resulted from LAD ligation. Scar size calculated as the percentage of scarred LV tissue weight versus total LV tissue weight was also comparable between the MI-induced groups (Figure 1E. 25.38 ± 1.42, vs. 21.50 ± 0.69, 20.54 ± 1.62, 20.51 ± 1.08, 22.06 ± 1.92).

### 2.2. Treatment with Resveratrol, Sacubitril/Valsartan, Valsartan and Sacubitril/Valsartan + Resveratrol Prevents Post-MI LV Dilatation 

**Figure 2:**
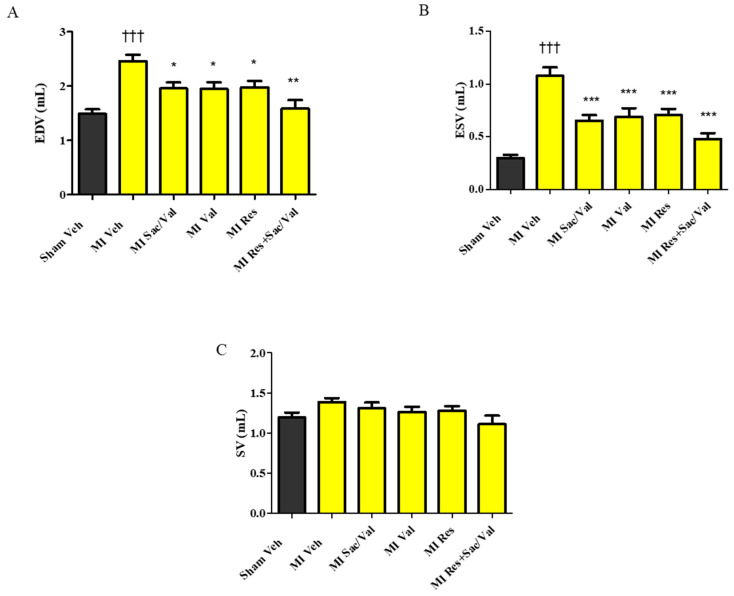
(**A**) End diastolic volume (EDV); (**B**) End systolic volume (ESV); (**C**) Systolic volume (SV). All values are expressed as mean ± SEM, *n* = 9–11. ^†††^ *p* < 0.001 vs. Sham Veh; * *p* < 0.05, ** *p* < 0.01, *** *p* < 0.001 vs. MI Veh. Veh—vehicle, Sac/Val—sacubitril/valsartan, Res—resveratrol, Val—valsartan.

Echocardiographic analysis showed that MI-induced rats treated with vehicle had significantly increased LV dilatation as evidenced by the increased LVID (Table 1. LVID diastole: 10.66 ± 0.18 vs. 8.86 ± 0.19, *p* < 0.001; LVID systole: 7.57 ± 0.31 vs. 4.93 ± 0.19, *p* < 0.001) when compared with sham-operated rats treated with vehicle at 8 weeks. As compared to MI-induced rats treated with vehicle, treatment with sacubitril/valsartan, valsartan, resveratrol and sacubitril/valsartan + resveratrol significantly decreased LV dilatation in MI-induced rats (Table 1. LVID diastole: 10.66 ± 0.18 vs. 9.78 ± 0.20, 9.85 ± 0.23, 9.82 ± 0.21, *p* < 0.05 and 9.01 ± 0.31, *p* < 0.001; LVID systole: 7.57 ± 0.31 vs. 6.54 ± 0.18, *p* < 0.05, 6.59 ± 0.30, *p* < 0.01, 6.53 ± 0.27, *p* < 0.05 and 5.81 ± 0.25, *p* < 0.001). LVPWT diastole and LVPWT systole were comparable between the groups. MI-induced rats treated with vehicle had significantly increased EDV and ESV when compared with sham-operated rats treated with vehicle at 8 weeks. In comparison to MI-induced rats treated with vehicle, MI-induced rats treated with sacubitril/valsartan, valsartan, resveratrol and sacubitril/valsartan + resveratrol had significantly lower EDV and ESV (Figure 2A,B). SV was comparable between the groups (Figure 2C).

### 2.3. Treatment with Resveratrol, Sacubitril/Valsartan, Valsartan and Sacubitril/Valsartan+ Resveratrol Prevents Post-MI Cardiac Dysfunction

**Figure 3:**
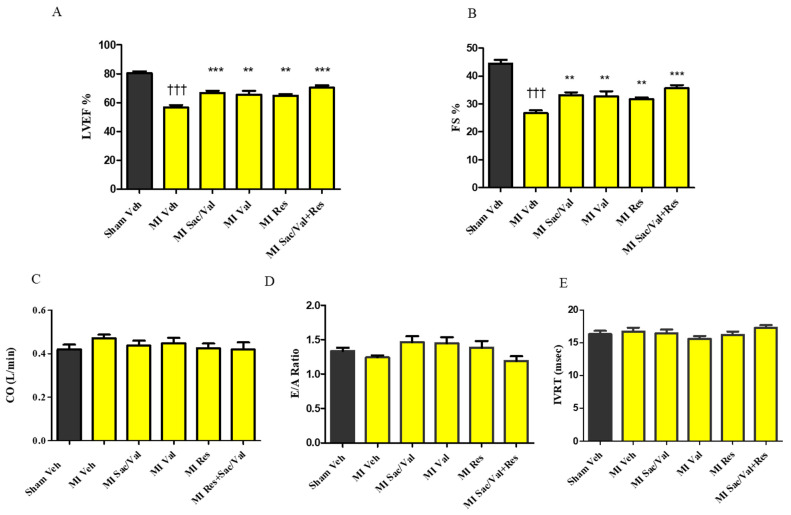
(**A**) Left ventricular ejection fraction (LVEF); (**B**) Fractional shortening (FS); (**C**) Cardiac output (CO); (**D**) E/A ratio; (**E**) Isovolumic relaxation time (IVRT) of sham and MI rats. All values are expressed as mean ± SEM, *n* = 9–11. ^†††^ *p* < 0.001 vs. Sham Veh; ** *p* < 0.01, *** *p* < 0.001 vs. MI Veh. Veh—vehicle, Sac/Val—sacubitril/valsartan, Res—resveratrol, Val—valsartan.

LVEF was significantly lower in vehicle-treated MI-induced rats compared to vehicle-treated sham-operated rats at 8 weeks (Figure 3A. 56.60 ± 1.70 vs. 80.33 ± 1.41, *p* < 0.001). In comparison to vehicle-treated MI-induced rats, sacubitril/valsartan, valsartan, resveratrol and sacubitril/valsartan + resveratrol treated MI-induced rats had significant improvements in LVEF (56.60 ± 1.70 vs. 66.82 ± 1.43, *p* < 0.001, 65.45 ± 2.70, 64.82 ± 1.02, *p* < 0.01 and 70.30 ± 1.63, *p* < 0.001).

FS was also significantly lower in vehicle-treated MI-induced rats compared to vehicle-treated sham-operated rats at 8 weeks (Figure 3B. 44.44 ± 1.36 vs. 26.70 ± 1.02, *p* < 0.001). In comparison to vehicle-treated MI-induced rats, sacubitril/valsartan, valsartan, resveratrol and sacubitril/valsartan + resveratrol received MI-induced rats had significantly improved FS (Figure 3B. 26.70 ± 1.02 vs. 33.18 ± 0.99, *p* < 0.001, 32.64 ± 1.94, 31.64 ± 0.69, *p* < 0.01 and 35.60 ± 1.17, *p* < 0.001). CO, IVRT and E/A ratio were comparable between the groups (Figure 3C–E).

### 2.4. Treatment with Resveratrol, Sacubitril/Valsartan, Valsartan and Sacubitril/Valsartan + Resveratrol Lowers Post-MI Increase in MDA, TNF-α, Collagen, and BNP

**Figure 4:**
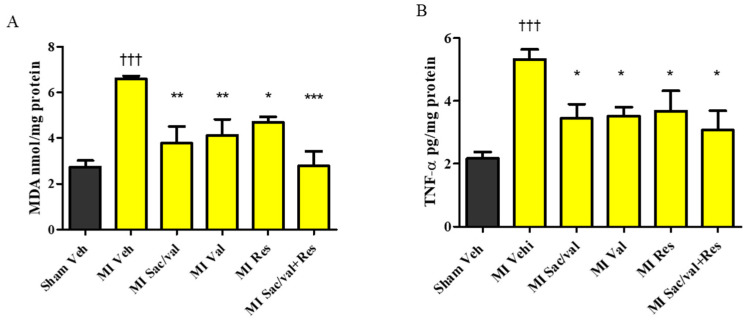
(**A**) Malondialdehyde (MDA); (**B**) Tumor necrosis factor-α (TNF-α). All values are expressed as mean ± SEM, *n* = 4–6. ^†††^ *p* < 0.001 vs. Sham Veh; * *p* < 0.05, ** *p* < 0.01, *** *p* < 0.001 vs. MI Veh. Veh—vehicle, Sac/Val—sacubitril/valsartan, Res—resveratrol, Val—valsartan.

**Figure 5:**
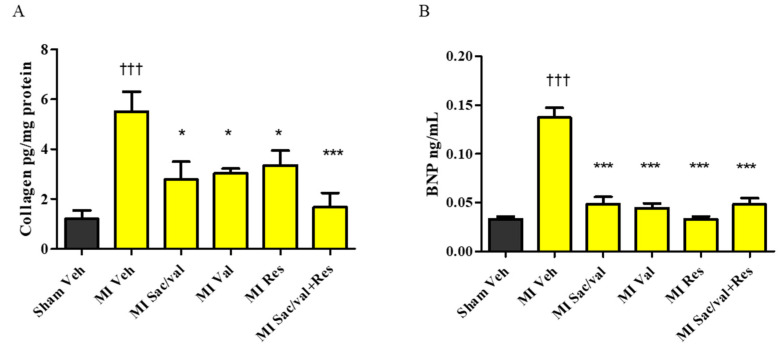
(**A**) Collagen; (**B**) brain natriuretic peptide (BNP). All values are expressed as mean ± SEM, *n* = 4–6. ^†††^ *p* < 0.001 vs. Sham Veh; * *p* < 0.05, *** *p* < 0.001 vs. MI Veh. Veh—vehicle, Sac/Val—sacubitril/valsartan, Res—resveratrol, Val—valsartan.

**Figure 6:**
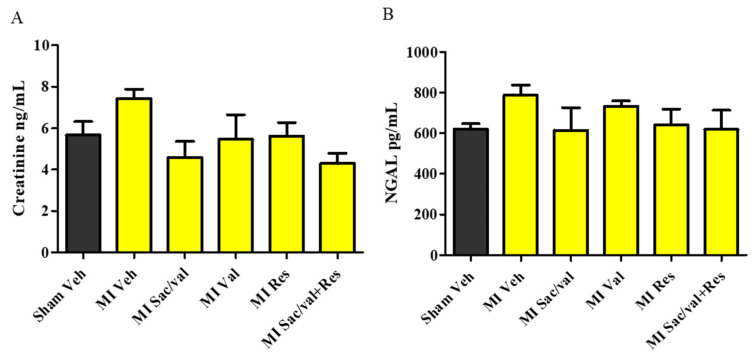
(**A**) Creatinine; (**B**) Neutrophil gelatinase associated lipocalin (NGAL) (plasma). All values are expressed as mean ± SEM, *n* = 4–6. Veh—vehicle, Sac/Val—sacubitril/valsartan, Res—resveratrol, Val—valsartan.

At 8 weeks, vehicle-treated MI-induced rats had significantly increased levels of MDA, a marker of oxidative stress, in LV compared with vehicle-treated sham-operated rats (Figure 4A). MI-induced rats treated with sacubitril/valsartan, valsartan, resveratrol and sacubitril/valsartan + resveratrol treatment had significantly lower levels of MDA compared to vehicle-treated MI-induced rats (Figure 4A. 6.59 ± 0.13 vs. 3.78 ± 0.72, 4.11 ± 71, *p* < 0.01, 4.69 ± 0.24, *p* < 0.05, and 2.78 ± 0.64, *p* < 0.001). We also observed that MI-induced rats showed significantly increased levels of TNF-α in LV when compared with vehicle-treated sham-operated rats at 8 weeks. Sacubitril/valsartan, valsartan, resveratrol, and sacubitril/valsartan + resveratrol also significantly reduced the levels of TNF-α in MI-induced rats compared to MI-induced rats treated with vehicle (Figure 4B. 5.33±0.31 vs. 3.45 ± 0.45, 3.52 ± 0.28, 3.68 ± 0.64, and 3.07 ± 0.61, *p* < 0.05). At 8 weeks, vehicle-treated MI-induced rats had significantly increased levels of collagen in LV compared with vehicle-treated sham-operated rats. MI-induced rats treated with sacubitril/valsartan, valsartan, resveratrol, and sacubitril/valsartan + resveratrol had significantly decreased levels of collagen compared to MI-induced rats treated with vehicle (Figure 5A. 5.50 ± 0.80 vs. 2.78 ± 0.71, 3.09 ± 0.20, 3.35 ± 0.59, and 1.67 ± 0.58, *p* < 0.05). Plasma BNP level was significantly increased in vehicle-treated MI-induced rats compared with vehicle-treated sham-operated rats. Sacubitril/valsartan, valsartan, resveratrol, and sacubitril/valsartan + resveratrol treatments also significantly reduced the levels of BNP in MI-induced rats (Figure 5B. 0.14 ± 0.01 vs. 0.05 ± 0.007, 0.04 ± 0.005, 0.03 ± 0.002, and 0.05 ± 0.006, *p* < 0.001). Plasma creatinine and NGAL levels were comparable between the sham-operated and MI groups (Figure 6A,B).

## 3. Discussion

This study revealed that the stand-alone administration of resveratrol and sacubitril/valsartan that was commenced immediately after MI-induction and continued for 8 weeks significantly prevented maladaptive LV remodeling and dysfunction. The combination treatment with sacubitril/valsartan + resveratrol was also cardioprotective in MI-induced rats. However, no statistically significant incremental benefit was observed with the sacubitril/valsartan + resveratrol combination treatment, despite a clear trend towards a further reduction in cardiac remodeling and enhanced systolic function (≥4% increase in LVEF), than with individual treatments. The resveratrol and sacubitril/valsartan mediated cardioprotection was also comparable to that of valsartan in MI-induced rats.

Previously, we reported that resveratrol was as beneficial as perindopril, in an equal dose (2.5 mg/kg body weight/day) comparison study, in improving cardiac structural and functional abnormalities in MI-induced rats [12]. An earlier study showed that a 4-week treatment with sacubitril/valsartan, started 1 week after the induction of MI in rats, improved cardiac remodeling and dysfunction [14]. Another study reported that sacubitril/valsartan reduced cardiac rupture and mortality compared with enalapril at 24 h when treatment was started after a day of MI-induction [15]. Similarly, in an ischemia/reperfusion model, sacubitril/valsartan treatment started along with reperfusion was able to preserve LVEF at 72 h after an ischemic episode in rabbits [16]. However, the study designs in previous reports precluded our ability to understand the advantages of an earlier and longer term sacubitril/valsartan treatment on MI-related cardiac remodeling and dysfunction that leads to HF in a permanent LAD-ligated model [15,16]. Here, we investigated the effects of both sacubitril/valsartan and resveratrol alone and a combination of both interventions by addressing the pertinent unanswered questions mentioned above. There was a significant reduction in LV remodeling and an improvement in systolic dysfunction when MI-induced rats were immediately treated with sacubitril/valsartan or resveratrol alone and in combination for a longer duration. All MI-induced rats started receiving the treatment within an hour of LAD-ligation. No interference with reparative scar formation was seen with the treatments even though they were started early. The dose of sacubitril/valsartan was derived from the PARADIGM-HF trial by calculating the equivalent dose for rodents [9,14]. The doses of resveratrol and sacubitril/valsartan were also different in our study because there are no previous studies that reported the benefits of both agents at a same dose. 

Sacubitril/valsartan and low-dose resveratrol treatment significantly prevented LV dilatation post-MI. The combination treatment with sacubitril/valsartan + resveratrol also afforded protection against LV dilatation with higher significance than the individual treatments in MI-induced rats. This finding suggests that sacubitril/valsartan and resveratrol effectively reduce pathological LV remodeling when treatment is started early. Low-dose resveratrol treatment has also been shown to mediate its cardioprotective effects by reducing left atrial and LV remodeling in MI-induced rats with HF [17]. Importantly, high-dose resveratrol treatment has been shown to reverse LV dilatation (reverse remodeling) when treatment was started after significant remodeling had already occurred in MI animals [18]. The treatments did not result in a statistically significant reduction in scar size % in MI-induced rats even though there was a trend towards reduction. A previous study also reported that infarct size was not reduced by sacubitril/valsartan treatment in MI-induced animals [14]. This underscores that the anti-remodeling benefits we observed in our study were not due to an infarct-sparing effect of the treatments. However, it should be noted that resveratrol and sacubitril/valsartan have been shown to reduce the infarct size post-MI [16,18,19]. This discrepancy in benefits of infarct size reduction with treatment could be due to different species, ischemic procedure and techniques used to calculate infarct size in the studies. 

It is recognized that 50% of patients fail to demonstrate an improvement in LVEF following acute MI even after undergoing reperfusion and/or receiving optimal drug therapy [20,21,22]. Consistent with previous studies, our study demonstrated sacubitril/valsartan improved LVEF and FS in MI-induced rats and thereby provided protection against further LV dysfunction [14,23]. In the current study, low-dose resveratrol prevented the early post-MI deterioration of systolic function as evidenced by the increased LVEF and FS in MI-induced rats. Recently, another preclinical study reported the efficacy of resveratrol in improving systolic function in the setting of post-MI associated HF [17], which showed that 3 weeks after LAD-ligation, MI-induced rats had signs of HF (LVEF < 40%), and low-dose resveratrol treatment for 2 weeks (started 3 weeks after surgery and HF was established) improved LVEF. CO was unchanged in MI-induced rats, and treatments also did not result in any drastic changes in CO in MI-induced rats. In addition, stand-alone and combination treatments with sacubitril/valsartan and resveratrol prevented a mild increase in the surrogate markers of lung and liver congestion in MI-induced rats suggesting that treatments prevented signs of HF in MI-induced rats. 

MI can lead to an imbalance between reactive oxygen species (ROS) and antioxidants, and the progression of cardiac remodeling and HF [24]. Consistent with previous studies [25,26], the elevated levels of MDA observed in MI-induced rats further demonstrates the contribution of altered oxidant status to the post-MI cardiac impairment. The lower levels of MDA in MI-induced rats which received resveratrol, sacubitril/valsartan, valsartan and combination of sacubitril/valsartan + resveratrol suggest that the improvement in cardiac structure and function may be partly mediated through an improvement in the redox status. The combination of sacubitril/valsartan + resveratrol was slightly better in lowering the levels of MDA in MI-induced rats. In addition, previous studies demonstrated that treatment with resveratrol or RAAS inhibitors/blockers involves an improvement in the antioxidant status [27,28,29,30,31,32,33,34]. TNF-α has also been linked with a higher risk of HF by directly contributing to cardiac remodeling and dysfunction [35,36]. A post-MI increase in TNF-α is also associated with LV systolic dysfunction, microvascular injury, and progressive myocardial necrosis [37]. Consistent with previous reports [38,39], our study also showed that resveratrol alone as well as in combination prevented the increase of TNF-α in MI-induced rats. Hence, the reduction in proinflammatory cytokines may also be involved in the improvement in cardiac dysfunction in MI-induced rats. Altered collagen turnover involving its breakdown and synthesis post-MI leads to extra-cellular matrix degradation and cardiac fibrosis and contributes to the progression of LV remodeling, contractile dysfunction [40,41]. Resveratrol is also known to inhibit myofibroblast differentiation via altering the transforming growth factor-β (TGF-β)/Smad3 pathway and ROS/extracellular regulated kinase/TGF-β1/periostin pathway [42,43]. Resveratrol decreases TGF-β1-induced cardiac fibroblast proliferation and collagen secretion partly through the downregulation of miR-17 and Smad7 mRNA and protein expression [44]. Sacubitril/valsartan is also known to prevent cardiac fibroblast to cardiac myofibroblast activation, which results in cardiac fibrosis [45]. Our data shows that resveratrol and sacubitril/valsartan treatments may improve cardiac remodeling and function via their antifibrotic actions.

BNP is released by the myocardium due to volume overload [46]. Both short-term and long-term elevations of BNP after MI, have been shown to be associated with poorer prognosis [46]. The current study showed that treatment with resveratrol, sacubitril/valsartan, valsartan and the combination was associated with a lower level of BNP in MI-induced rats. Previous preclinical studies have also reported that resveratrol treatment effectively lowers the level of BNP [47,48,49]. Renal dysfunction is also considered as one of the independent risk factors for major cardiovascular events and mortality in post-MI patients [50]. However, we did not observe any structural or functional renal impairment in MI-induced rats as evidenced by the unaltered level of NGAL and creatinine, which are surrogate markers of kidney injury and function, respectively. 

Future perspectives: Considering that the combination treatment (sacubitril/valsartan + resveratrol) provided a slight incremental improvement in cardiac parameters (over resveratrol or sacubitril/valsartan alone) in the current study with low dose resveratrol, future studies may consider examining whether a higher dose of resveratrol (>2.5 mg/kg/day) along with sacubitril/valsartan 68 mg/kg/day) would act synergistically to provide a robust improvement in cardiac structure and function in post-MI rats. No adverse effects have been reported with 2.5 mg/kg body weight/day in rats (human equivalent 28 mg/70 kg human) [12,51]. Resveratrol up to 1 g/day has proved to be safe in humans in dose response studies [52,53]. As per the preclinical results in this study, an adjuvant therapy such as resveratrol may have potential benefits in post-MI patients when the current prescribed therapy is not well-tolerated. In this regard, sacubitril/valsartan is known to cause hypotension and an angiotensin receptor blocker (ARB) such as valsartan may also result in hypotension and renal complications due to RAAS blockade [9,54]. It should be noted that in the PARADIGM-HF trial, 12% of patients did not complete the run-in period and were not included in the study because of drug-induced adverse events [9]. Given the similar extent of cardioprotection observed in MI-induced rats with resveratrol and perindopril treatment in our previous study [12], and with resveratrol and valsartan in the current study, it may be useful to examine the possibility of resveratrol of being an inhibitor of the angiotensin II signaling pathway in future studies.

## 4. Materials and Methods

### 4.1. Animal Care and Experimental Design

This study protocol (16-002/1) was approved by the University of Manitoba Office of Research Ethics & Compliance and Animal Care Committee and all the procedures were done in accordance with the guidelines from the Canadian Council for Animal Care. Male Sprague Dawley rats (175–215 g) were first acclimatized and housed in a temperature and humidity controlled room with a 12-h light/dark cycle (Charles River Laboratories, Montreal, QC, Canada). For surgery, rats were anesthetized with 1–5% isoflurane with oxygen at a flow rate of 2 L/min and kept in the surgical plane on anesthesia with 2% isoflurane during surgery. They were subjected to permanent ligation of the left anterior descending artery (LAD) to induce MI or sham surgery after baseline echocardiographic examination. A left thoracotomy was performed, and the heart was accessed through an incision on pericardial sac. The LAD was traced and blocked with 6-0 polypropylene silk suture at a region 2 mm away the aortic root. Specifically, the suture was tied carefully, and the ligation was deemed effective once the anterior wall of the LV gradually turned pale. The heart was repositioned, the chest compressed to remove any air from the cavity and the incision was closed using a purse string suture. Sham-operated animals served as the control and were subjected to the same surgical procedure except that the LAD was not ligated. Buprenorphine 0.05 mg/kg was administered before and after surgery (2 times a day for 2 days) subcutaneously as an analgesic agent to all rats. All surviving sham and MI-induced rats were assigned to 5 different treatment groups. 1. Vehicle (50% ethanol 2.5 mL/kg body weight/day), 2. Sacubitril/valsartan (68 mg/kg body weight/day, Novartis, Basel, Switzerland), 3. Resveratrol (2.5 mg/kg body weight/day, trans-resveratrol, ≥99%, Sigma-Aldrich, Ltd., Oakville, ON, Canada), 4. Valsartan (31 mg/kg body weight/day, Novartis, Basel, Switzerland), 5. Sacubitril/valsartan + resveratrol (68 mg/kg body weight/day + 2.5 mg/kg body weight/day). One sham group and 1 MI group received vehicle treatment, whereas another 4 MI groups received respective investigational agents as treatment. All groups received the treatments by oral gavage daily for 8 weeks which was approved by the Research Ethics & Compliance and Animal Care Committee. The doses for the present study were chosen based on previous studies which showed cardiac benefits in same animal model with resveratrol [12,14]. Resveratrol was dissolved in 50% ethanol to ensure maximum absorption and efficacy and hence 50% ethanol was used as a vehicle, same as in our previous study [12]. No adverse effects were observed in that study with ethanol treatment in normal sham rats or MI-induced rats that received resveratrol dissolved in ethanol [12]. Animals were regularly weighed, and evaluated for well-being throughout the study.

### 4.2. Transthoracic Echocardiography (TTE)

Rats were weighed and anaesthetized with 3% isoflurane in a chamber, and then kept under 1.5–2% isoflurane throughout the procedure. TTE was obtained at baseline and at 8 weeks of treatment by 2D guided M-mode and Doppler modalities with a 13-MHz probe (Vivid E9; GE Medical Systems, Milwaukee, WI) by a procedure described elsewhere [55]. Two-D M-mode parasternal short-axis view images were obtained to measure LV internal diameter (LVID), LV posterior wall thickness (LVPWT), end diastolic volume (EDV), end systolic volume (ESV), stroke volume (SV) and left ventricular ejection fraction (LVEF), fractional shortening (FS) and cardiac output (CO). Doppler measurements included measurement of isovolumic relaxation time (IVRT) and early wave/after (E/A) ratio. All images were analyzed using EchoPAC software (GE Medical Systems, Milwaukee, WI, USA). The values obtained for the mentioned parameters in 3 consecutive cardiac cycles were averaged to obtain the final data [55,56]. 

### 4.3. Blood and Tissue Collection

All animals were anesthetized with 1–5% isoflurane. Depth of anesthesia was assessed by pedal withdrawal reflex. The blood sample was drawn from the inferior vena cava by opening the thoracic cavity. After blood collection, the heart was immediately excised, rinsed in PBS and atria, right and LV, septum, as well as, fibrotic scar tissue separated, weighed, flash frozen in liquid nitrogen and stored at −80 °C. Lungs and liver were also collected.

### 4.4. LV Scar Size and Lung and Liver Wet-to-Dry Weight Ratio Determination

The percentage of scarred (infarcted) LV tissue was calculated by dividing the weight of scarred LV tissue by whole weight of LV tissue [57]. Evidence of HF was assessed by checking for the presence of ascites in abdomen, and by calculating lung wet-to-dry weight ratio and liver wet-to-dry weight.

### 4.5. Oxidative Stress Marker Assay

The level of the lipid peroxidation product, malondialdehyde (MDA), in the LV was assessed using the MDA quantification kit (Abcam, Cambridge, UK) following kit manufacturer’s instructions [58]. 

### 4.6. Proinflammatory and Cardiac Fibrosis Marker Assays

The levels of tumor necrosis factor-α (TNF-α) and hydroxyproline in the LV were assayed following the kit manufacturer’s instructions (Abcam, Cambridge, UK). Collagen concentration was calculated by multiplying the hydroxyproline level by a factor 7.46 as the interstitial collagen contains an approximately 13.4% hydroxyproline by a procedure described elsewhere [57].

### 4.7. NP and Renal Dysfunction and Injury Marker Assays

The levels of plasma BNP, creatinine and neutrophil gelatinase associated lipocalin (*NGAL*) were also measured as per kit manufacturer’s instructions (Abcam, Cambridge, UK).

### 4.8. Statistical Analysis

All values are expressed as means ± SEM. One-way analysis of variance was used to analyze variations between the means of the groups. Significant values are defined as *p* < 0.05. If a significant difference was observed, one-way analysis of variance was followed by a Newman-Keuls post hoc test.

## 5. Conclusions

Stand-alone treatment with resveratrol and sacubitril/valsartan significantly prevented cardiac remodeling and dysfunction in MI-induced rats. The combination treatment with sacubitril/valsartan + resveratrol also prevented cardiac abnormalities with slightly better protection. Resveratrol, sacubitril/valsartan, valsartan, and sacubitril/valsartan + resveratrol mediated prevention in cardiac remodeling and dysfunction was partly mediated by a reduction in cardiac oxidative stress, inflammation and fibrosis. There was no evidence of renal dysfunction or injury after MI. Our results suggest that sacubitril/valsartan and resveratrol are beneficial and may be further explored for their clinical efficacy in the setting of MI in future clinical trials.

## Figures and Tables

**Table 1:** Left ventricular internal diameter (LVIDd and LVIDs), and left ventricular posterior wall thickness (LVPWTd and LVPWTs) at diastole and systole of sham and MI rats. All values are expressed as mean ± SEM, *n* = 9–11. ^†††^ *p* < 0.001 vs. Sham Veh; * *p* < 0.05, ** *p* < 0.01, *** *p* < 0.001 vs. MI Veh.

	Sham Veh	MI Veh	MI Sac/Val	MI Val	MI Res	MI Sac/Val + Res
**LVIDd** **(mm)**	8.86 ± 0.19	10.66 ± 0.18 ^†††^	9.78 ± 0.20 *	9.85 ± 0.23 *	9.82 ± 0.21 *	9.01 ± 0.31 ***
**LVIDs ** **(mm)**	4.93 ± 0.19	7.57 ± 0.31 ^†††^	6.54 ± 0.19 *	6.59 ± 0.30 **	6.53 ± 0.27 *	5.81 ± 0.25 ***
**LVPWTd (mm)**	1.94 ± 0.13	2.28 ± 0.09	2.02 ± 0.12	2.33 ± 0.07	2.21 ± 0.12	2.31 ± 0.06
**LVPWTs (mm)**	3.03 ± 0.16	2.99 ± 0.11	2.80 ± 0.16	3.14 ± 0.09	3.01 ± 0.15	3.14 ± 0.10

## Data Availability

The data presented in this study are available on reasonable request from the corresponding author.
